# Corrosion Behavior of Ti60 Alloy under a Solid NaCl Deposit in Wet Oxygen Flow at 600 °C

**DOI:** 10.1038/srep29019

**Published:** 2016-06-30

**Authors:** Lei Fan, Li Liu, Zhongfen Yu, Min Cao, Ying Li, Fuhui Wang

**Affiliations:** 1 Institute of Metal Research, Chinese Academy of Sciences, Wencui Road 62, Shenyang 110016, Liaoning, China; 2 Key Laboratory for Anisotropy and Texture of Materials (MoE), School of Materials Science and Engineering, Northeastern University, NO. 3-11, Wenhua Road, Heping District, Shenyang 110819, Liaoning, China

## Abstract

The corrosion behavior of Ti60 alloy covered with a solid NaCl deposit in wet oxygen flow at 600 °C has been studied further by SEM, EDX, XPS, XRD, TEM and EPMA analysis. The results show that solid NaCl and H_2_O react with Ti oxides, which destroyed the Ti oxide scale to yield the non-protective Na_4_Ti_5_O_12_ and other volatile species. The resulting corrosion product scale was multilayered and contained abundant rapid diffusion channels leading to the fast diffusion which improved the corrosion rate. A possible mechanism has been proposed for the NaCl-covered Ti60 alloy, based on the experimental results.

It is widely known that the turbine blades of airplanes or ships suffer considerably more severe corrosion when deployed in service in marine environments, compared to inland areas. This is mainly attributable to the presence of moist, salt-rich air in marine environments, especially NaCl, and water vapor. The turbine blades often operate at intermediate temperatures (around 300–600 °C), wherein NaCl is in a solid state, hence solid NaCl becomes deposited on the blade surface. This solid NaCl deposit interacts synergistically with the moist air to further enhance the corrosion rate^1^,^2^,^3^,^4^,^5^,^6^,^7^.

The corrosion behavior of several traditional blade metals or alloys has been extensively studied, including pure Cr^1^,^2^,^3^, Fe-Cr alloys^4^, 1Cr11NiW2MoV steel^5^, 304 stainless steel^6^ and K38G alloy^7^. Also, Liu *et al*.^8^ reviewed the corrosion behavior of Fe-Cr alloys under the solid NaCl at 400–700 °C. The corrosion rate of these materials is sensitive to the presence of the NaCl deposit which reacts with and destroys the protective metal surface oxide scale. Furthermore, Tang *et al*.^3^,^9^ reported that the whole corrosion process of the pure Fe and pure Cr contained electrochemical reactions which coupled with a preceding chemical reaction process (ce) to accelerate the corrosion process. Tang *et al*.^10^ also found the similar corrosion of pure Fe under a solid Na_2_SO_4_ deposit layer in wet O_2_ flow. However, the increase effect of Na_2_SO_4_ for the corrosion is less than that of solid NaCl. All of above studies focused on the corrosion behavior of Fe-Cr alloys under this special environment. The results showed that the corrosion resistance of Fe-Cr alloy was weak, and new more anticorrosion materials should be applied, especially under this severe environment.

Near-alpha titanium alloys are considered as materials for compressor blades due to their superior strength/weight ratio, good creep resistance and excellent mechanical properties^11^,^12^. Currently, the mechanical properties of titanium alloys have been sufficiently optimized to virtually satisfy the requirements for compressor blades. However, the strong affinity between titanium alloys and oxygen has raised serious corrosion concerns, which have notably hindered the use of titanium alloys in certain applications^13^,^14^. Additionally, Yao *et al*.^15^ observed the corrosion of titanium alloys to be seriously accelerated by solid NaCl deposit, even in amounts as low as 2.3 × 10^−2^ mg/cm^2^. Such NaCl deposit induced effects have been shown to be further aggravated by the presence of water vapor^16^, which raises additional concerns about the suitability of such alloy for marine-based applications, under conditions that promote formation of solid NaCl. Thus, it is essential to assess the corrosion behavior of titanium alloys in marine environments at intermediate temperatures.

Ti60 alloy, a near-alpha titanium alloy, is considered as a fallback material for the fabrication of compressor blades. In previous studies, the corrosion behavior of Ti60 alloy covered by a solid NaCl deposit in wet oxygen flow at 500–700 °C was investigated^17^. The results revealed that the corrosion of Ti60 alloy was severe and a non-protective, thick and complex corrosion product layer was formed in the presence of solid NaCl. The fast corrosion was ascribable to a reaction of solid NaCl and Ti oxide forming on the surface. However, the relevant mentioned products of the reaction (Na_2_TiO_3_, HCl and Cl_2_) in the inferred mechanism have never been identified in the experiment. Additionally, the morphologies of the corrosion product scale is not studied unmistakably. Thus, the corrosion behavior has not been clarified up to now and the exact mechanism of the process remains is unclear.

In this paper, the corrosion behavior of Ti60 alloy underneath a solid NaCl deposit has been investigated in wet oxygen flow at 600 °C. The compositions and the morphologies of the corrosion products were studied in detail using scanning electron microscopy (SEM) equipped with an energy dispersive spectrometer (EDS), electron probe microanalysis (EPMA), transmission electron microscopy (TEM), X-ray photoelectron spectroscopy (XPS) and X-ray diffraction (XRD). Possible mechanisms for the associated reactions and processes have been discussed based on the experimental results.

## Results

### Corrosion kinetics

**Figure 1:**
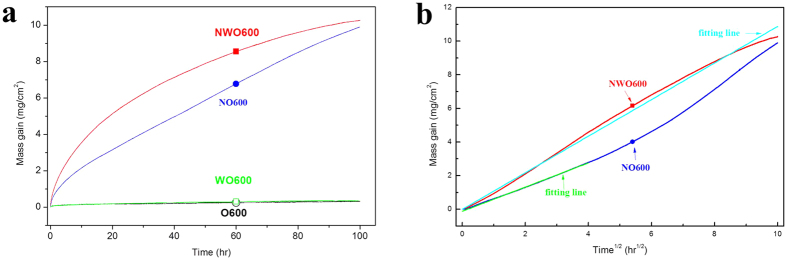
Corrosion kinetics curves (**a**) and mass gain *vs* t^1/2^ plot (**b**) of Ti60 alloy under different corrosion environments at 600 °C: (I) dry O_2_ (—○—), (II) H_2_O + O_2_ (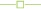), (III) NaCl + O_2_ (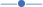), (IV) NaCl+ H_2_O + O_2_ (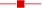).

Figure 1a shows the corrosion kinetics of Ti60 alloy under the different test environments at 600 °C. It is evident that the weight of samples in the pure O_2_ environment (O600) and wet O_2_ (WO600) changed little during the testing time, which suggests that the corrosion of Ti60 alloy in these environments was not very significant in the absence of the NaCl deposit. However, the mass gain is about two orders of magnitude larger in NaCl+O_2_ (NO600) and NaCl+H_2_O+O_2_ (NWO600) than in O600 and WO600, due to the presence of the solid NaCl deposit. This means that Ti60 alloy suffered severe corrosion due to the presence of the NaCl deposit at 600 °C. Furthermore, it appears that the corrosion in NWO600 was more serious than in NO600, especially at the earlier stages (0–20 hr), which indicates that water vapor further accelerated the corrosion process under these conditions. This implies synergistic interactions between the solid NaCl deposit and water vapor. These results are similar to that reported for Fe-Cr alloys under the similar conditions^5^.

As shown in Fig. 1b, the plot of mass gain *vs* t^1/2^ follows a linear relationship in NWO600; thus, the corresponding corrosion kinetics obeys parabolic rate law. For the Ti60 alloy in NO600, the linear relationship is only observed at the initial stages (0–20 hr), and thereafter deviates from linearity, i.e. from 20 to 100 hr. Thus, the corrosion kinetics obeys parabolic rate law only at the initial stages. In addition, the kinetics curves for long time corrosion (20–100 hr) in NO600 obeys the linear rate law (as shown in Fig. 1a), and the corresponding corrosion is a chemical reaction rate determining step. Hence, when exposed in NO600, the corrosion kinetics obeys parabolic rate law followed by a chemical reaction rate determining corrosion process.

### Morphologies and compositions of solid corrosion products

**Figure 2:**
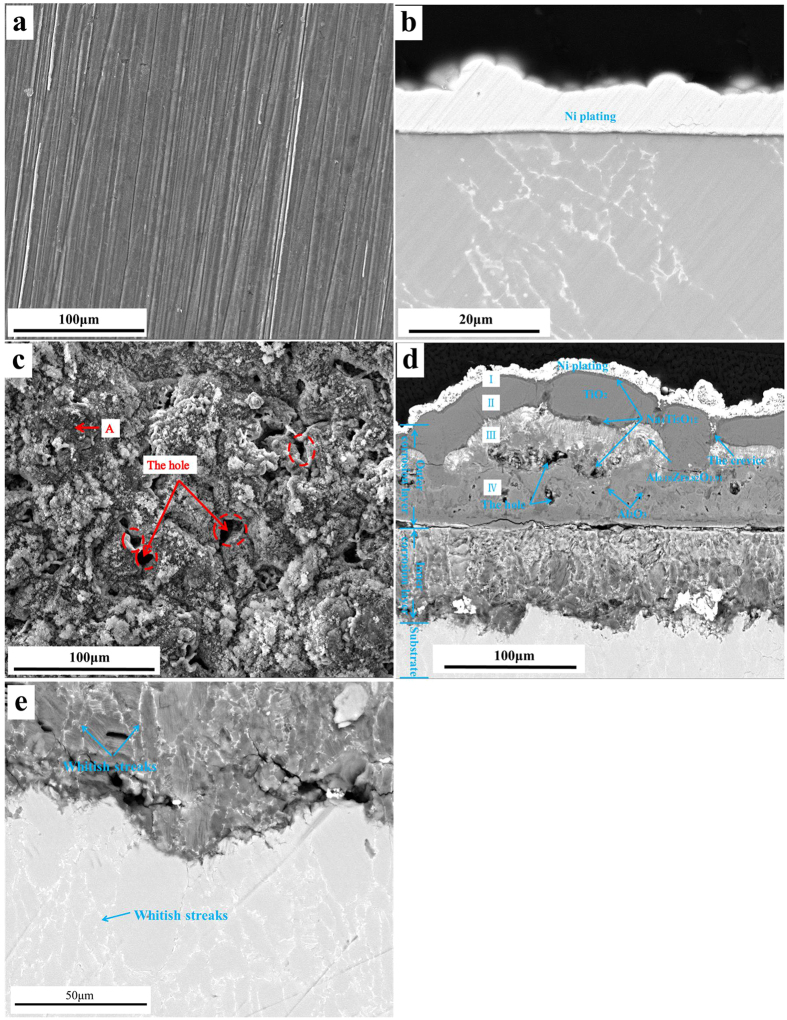
Surface morphologies (**a**) and cross-sectional morphologies (**b**) of Ti60 alloy after 100 hr expourse in O_2_ at 600 °C; and surface morphologies (**c**) and cross-sectional morphologies (**d**) of Ti60 alloy under a NaCl deposit in H_2_O + O_2_ at 600 °C for 100 hr and higher magnification image (**e**) of the inner corrosion layer.

Figure 2 shows the surface morphologies of the Ti60 alloy after 100 hr exposure in (a) O600 and (c) NWO600. Figure 2a shows a virtually oxide-free surface for the specimen in O600, with visible scratch lines (produced by mechanical abrasion during the preparation of specimen). On the contrary, an ample and porous layer of corrosion product was formed after 100 hr exposure in NWO600 as shown in Fig. 2c. The corrosion products include both compact (region A) and the loose parts (region B).

After corrosion, the samples were wrapped into a thin nickel foil to protect the oxide scale from fracture and spall during the metallographic preparation. Then, they were embedded into the epoxy resin, ground to 5000 grit with SiC paper and finally polished with diamond paste for cross-sectional observation by SEM. Figure 2b present the cross-sectional morphologies of the Ti60 alloy after 100 hr exposure in O600. The micrographs show that a very thin, compact and continuous scale was formed on the Ti60 alloy after 100 hr exposure in pure O_2_ flow. Contrariwise, a thick (~180 μm), multilayered and complex corrosion scale was observed on the Ti60 alloy specimen exposed in NWO600 for 100 hr (Fig. 2d). The complex corrosion scale comprised an outer corrosion layer (OCL) and an inner corrosion layer (ICL). The thickness of the OCL varied between 80 μm and 120 μm while that of the ICL was about 80 μm. Furthermore, the complicated OCL is divided into different parts as clearly illustrated in Fig. 2d.

**Figure 3:**
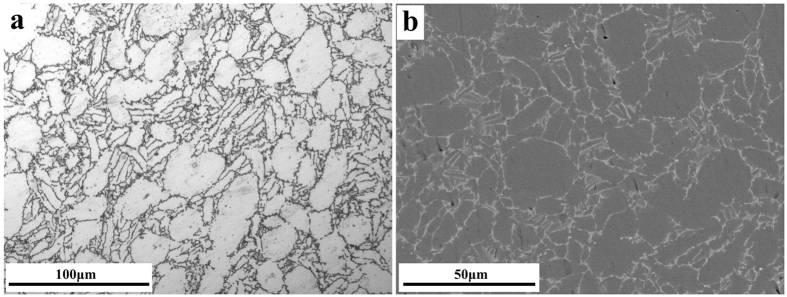
Metallographic (**a**) and backscattered electron image (**b**) of Ti60 alloy.

In order to investigate the microstructure around the substrate/ICL interface in detail, a higher magnification image was obtained (Fig. 2e), which revealed that the ICL had a layered structure. Also, the micrograph reveals a whitish streak (marked in Fig. 2e by blue arrows) traversing both the substrate and the ICL. EDS results (not shown) show non uniform distribution of elements, with Zr, Mo, Nb and Ta enriched in the whitish streaks. Interestingly, similar features of elemental enrichment and microstructure of the whitish streaks were also observed in the backscattered electron image of the uncorroded Ti60 alloy (Fig. 3b). Thus, the whitish streaks should be the inherent microstructure for the Ti60 alloy. During corrosion, the β stabilizing elements (Mo, Nb and Ta) are difficult to diffuse or transfer in the ICL. Thus, the non-uniform distribution of elements is a genetic microstructure during corrosion process.

**Figure 4:**
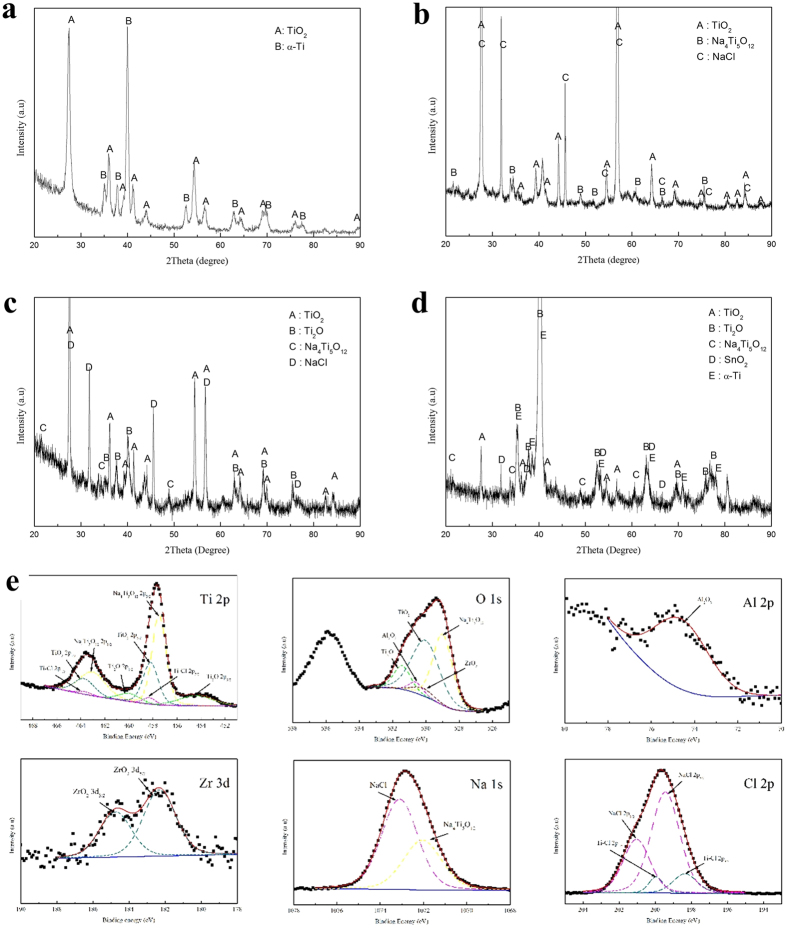
X-ray diffraction (XRD) patterns of Ti60 alloy exposed in O_2_ up to 100 hr at 600 °C (**a**) and under a NaCl deposit in H_2_O + O_2_ at 600 °C for 100 hr: (**b**) the patterns of surface products, (**c**) the patterns of the powder products of all the corrosion product scale, (**d**) the patterns of the inner corrosion layer products. X-ray photoelectron spectroscopy (XPS) spectra (**e**) for Ti, O, Al, Zr, Na and Cl of the corrosion products of Ti60 alloy after being exposed in NWO600 for 20 hr.

Figure 4 shows the XRD patterns of the corrosion products. From Fig. 4a, only TiO_2_ was formed on the surface of Ti60 alloy on exposure to O600 for 100 hr, which is in agreement with^11^,^14^,^18^,^19^. The XRD patterns in Fig. 4b show a mixture of corrosion products (TiO_2_ and Na_4_Ti_5_O_12_) on the surface of Ti60 alloy after exposure in NWO600 for 100 hr, with the residual NaCl still observed on the surface. Since the thickness of the corrosion scale on Ti60 alloy exposed in NWO600 for 100 hr is much larger than the detection depth of the XRD machine, the powders of the corrosion products scratched from the specimens after corrosion were analyzed to identify the chemical constituents of the corrosion scales. The results, as presented in Fig. 4c, show the corrosion products to be mainly composed of TiO_2_, Ti_2_O, Na_4_Ti_5_O_12_ and residual NaCl. The composition of the ICL was similarly analyzed after removing the OCL by mechanical polishing. The results in Fig. 4d show the constituents of the ICL to be TiO_2_, Ti_2_O, Na_4_Ti_5_O_12_, and SnO_2_. Some α-Ti was also observed, which may be from the substrate.

The XPS patterns of the corrosion products are also presented in Fig. 4e. The identification of peaks was performed by reference to an XPS database. The fitting results show that the corrosion products mainly compose Ti oxides (TiO_2_ and Ti_2_O), Na_4_Ti_5_O_12_, Al oxides and Zr oxides, with the residual NaCl.

### Cross-sectional distributions of elements in the corrosion products

**Figure 5:**
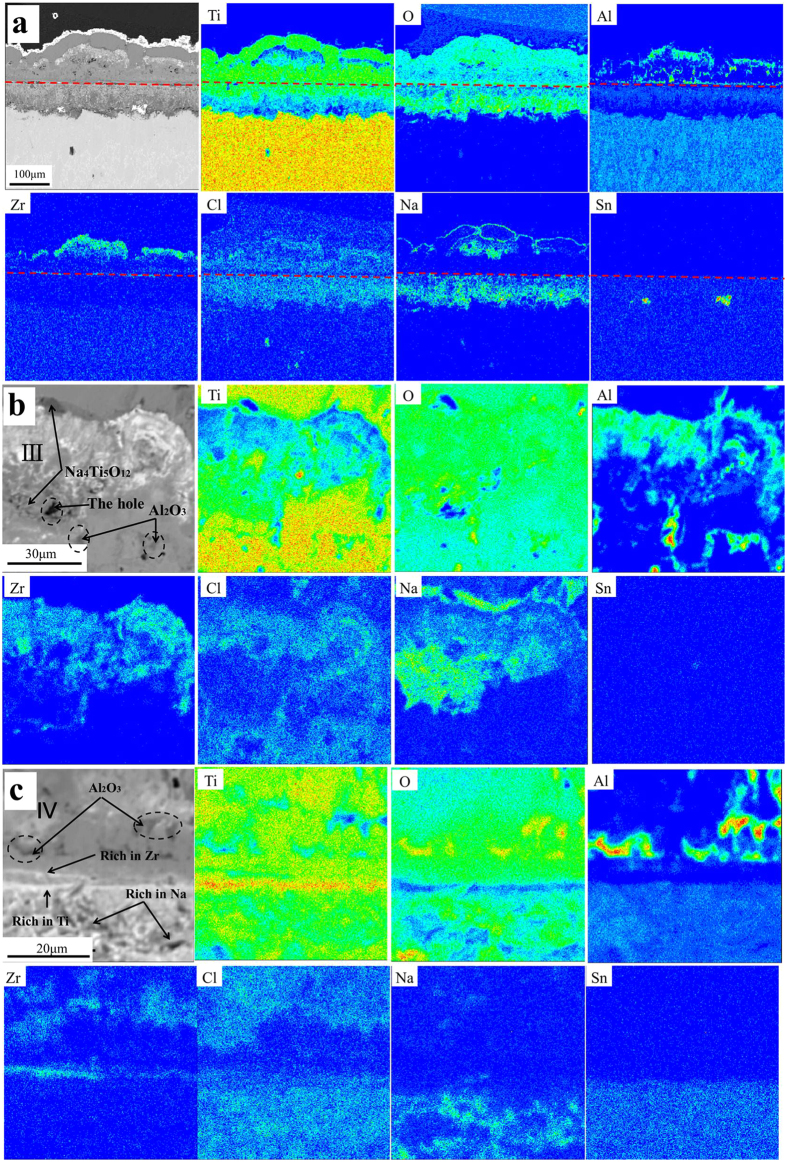
Electron probe microanalysis (EPMA) maps of elemental distribution on the cross-section of Ti60 alloy under a NaCl deposit in H_2_O + O_2_ at 600 °C for 100 hr (**a**); and the cross-sectional element analysis by EPMA of the part III (**b**) and the interface between the outer and the inner corrosion layer (**c**) of Ti60 alloy under a NaCl deposit in H_2_O + O_2_ at 600 °C for 100 hr.

Figure 5a shows the maps of the elemental distribution on the Ti60 alloy after 100 hr exposure in NWO600 and indicate that Ti, O and Cl were almost disperse all over the corrosion scales. Specifically, Ti was minutely present in part III of the OCL as well as the inner part of the ICL. Cl enrichment occurred in part III, part IV and especially the ICL, with O enrichment in part III and the inner part of the ICL. In contrast, the distribution of Al, Zr, Na and Sn was non uniform. Al enrichment was observed in part III and part IV of the OCL, while Zr was enriched in part III and slightly enriched at the OCL/ICL interface. In a word, part III is rich in Al, Zr and O and poor in Ti, which may be Al-Zr mixed oxide. Sn was slightly enriched in the ICL (forming the SnO_2_ shown in Fig. 4d) but was almost nonexistent in the OCL. This means that Sn did not diffuse outwards during the corrosion. To sum up, O, Na and Cl diffuse inwards to the ICL and react with the metal, while Ti, Al, and Zr diffuse outwards and form corrosion products in the OCL.

From the results in Fig. 5a, it is obvious that part III of the OCL and the OCL/ICL interface are complicated. They were thus further analyzed at higher magnification by EPMA and the results are presented in Fig. 5b,c respectively. There is clear enrichment of Al and Zr in part III in the form of Al-Zr mixed oxides. Besides, enrichment of Na (Na_4_Ti_5_O_12_) is obvious at the top region in addition to the loose region in part III, with minimal Cl presence. Slight enrichment of Cl is as well obvious in part III, with the Al-Zr mixed oxides. In Fig. 5c, Al and O are discontinuously enriched in part IV of the OCL and enriched in the region where Ti is relatively poor; thus, it is identified as Al_2_O_3_.

### TEM investigation of the ICL

In order to observe the microstructure of the ICL in detail, TEM analysis was undertaken. The cross-sectional TEM specimens were prepared as described in^20^,^21^. Firstly, the OCL was removed from the specimens after corrosion by mechanical polishing. Second, the specimens now having the ICL were cut into rectangles (3 mm × 1.5 mm × 0.7 mm) and glued together with face to face with an adhesive. The specimens were then mechanically polished to a thickness of approximately 50 μm and glued onto a 3-mm copper ring. Finally, the specimens were dimpled to a thickness less than 20 μm and thinned by argon ion beam.

**Figure 6:**
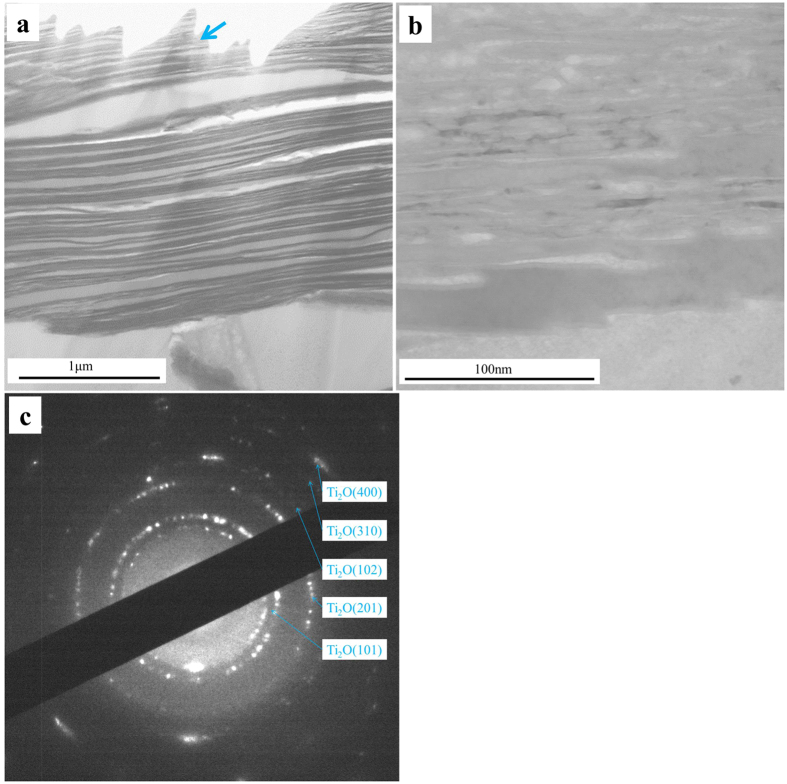
Cross-sectional STEM morphologies (**a**), high magnification image (**b**) and SAED patterns (**c**) of the inner corrosion layer of Ti60 alloy under a NaCl deposit in H_2_O + O_2_ at 600 °C for 20 hr.

Figure 6 presents the STEM (TEM in scanning mode) image of the ICL of the Ti60 alloy after being exposed in NWO600 for 20 hr. A layered structure is clearly observed from Fig. 6a. The high magnification image of a single layer from Fig. 6a (arrowed) presented in Fig. 6b indicates a lath-like structure. The selected area electron diffraction (SAED) technique was employed to determine the crystal structure of the ICL. The ringed SAED patterns, as shown in Fig. 6c, indicate that the ICL is polycrystalline, precisely Ti_2_O, as shown.

**Figure 7:**
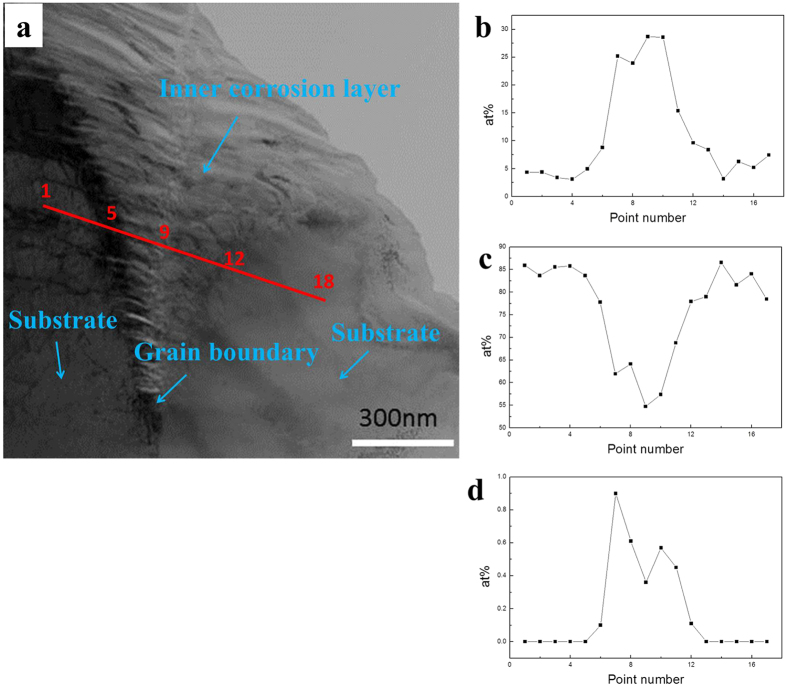
Cross-sectional STEM morphologies of the interface between the inner corrosion layer and the substrate of Ti60 alloy under a NaCl deposit in H_2_O + O_2_ at 600 °C for 20 hr (**a**), and the elemental distribution (**b–d**) of several elements (O, Ti, Cl) along the red line in Fig. 7a: (**b**) is the elemental distribution of O, (**c**) is the elemental distribution of Ti and (**d**) is the elemental distribution of Cl.

Figure 7a shows the STEM image of the ICL/substrate interface of the Ti60 alloy after being exposed in NWO600 for 20 hr. The distributions of O, Ti and Cl along the red line curve on Fig. 7a was investigated and the results shown in Fig. 7. The regions around point 1 and point 18 are within the alloy substrate, where O is poor and metallic elements are rich. The region from point 5 to point 12 is obviously the ICL, from the O enrichment in that area. The region around point 9 represents the original grain boundary of the substrate. The line distribution of Cl indicates that Cl is enriched in the ICL around the original gain boundary; hence, Cl preferentially diffuses into the substrate and reacts with metal along the grain boundaries.

## Discussion

The corrosion kinetics plots (Fig. 1) and the morphologies of corrosion products (Fig. 2a,b) show no significant corrosion for the Ti60 alloy specimen exposed in O600 or WO600, due essentially to the formation of a compact and protective TiO_2_ scale on the surface (Fig. 4a) through the reaction of Ti and oxygen or water vapor.


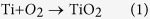



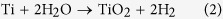


However, specimens exposed in the presence of solid NaCl (NO600 and NWO600) suffered severe corrosion (Fig. 1) and the resultant corrosion product scale was thick, porous and highly complex and layered structure (Fig. 2c,d). Thus, the corrosion of Ti60 alloy is greatly accelerated by solid NaCl deposit. From the corrosion kinetics (Fig. 1), the corrosion in NWO600 obeys parabolic rate law, and the diffusion is the rate determining process for the whole corrosion.

In the OCL, the abundant macro structural defects provide rapid diffusion channels for corrosive species (for example, oxygen, chlorine and water vapor)^1^,^4^,^5^,^7^,^17^. In the layer-structured ICL, the layer spacing could serve as high diffusion path for ionic species, due to the presence of defects and also high energy. Thus, the layer structure accelerates the outward diffusion of metal ions as well as the inward penetration of Cl ions and O ions. Also, the polycrystalline oxides on the ICL contain lots of grain boundaries, which could also further facilitate the diffusion of ionic species. The ICL is also enriched in Cl (Fig. 5), but our XRD results (Fig. 4) did not directly identify the presence of MCl_x_ as shown in Fig. 4d (M = metal). Interestingly, our XPS results (Fig. 4e) identify the presence of Ti-Cl bond and Luo *et al*.^22^ observed that Cl could partially replace some O in TiO_2_. Accordingly, we propose that Cl partially substitutes O in Ti oxide to form 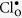 in the ICL. The Cl doping within the ICL is considered as a defect and can provide additional acceleration to the diffusion of ions in the corrosion layer. All of these effects contribute to the rapid diffusion of ionic species in the ICL.

For the alloy substrate, the grain boundaries could serve as high diffusivity paths^23^,^24^,^25^,^26^ due to their high energy, and thereby allow the alloy elements to travel to the surface during high temperature corrosion^26^. Also, the corrosive species (such as oxygen and chlorine) diffuse to the substrate quickly along the grain boundaries of original alloy as shown in Fig. 7 and react with the substrate metals.

In conclusion, the diffusion in the corrosion product scale (including the OCL and the ICL) is rapid during the corrosion; thus, the corrosion scale (including the OCL and the ICL) becomes essentially non protective beneath the NaCl deposit and the corrosion rate is greatly accelerated. Additionally, based on the fast diffusion phenomena highlighted above, we propose the following corrosion mechanism for the Ti60 alloy specimen exposed in NWO600:

The main constituents of the corrosion products (Fig. 4d) are Ti oxides (TiO_2_ and Ti_2_O), since during the corrosion, Ti diffuses outward and reacts with oxygen and water vapor, which diffuse inward quickly through the macro defects, to form TiO_2_ as described in reaction (1) and reaction (2). Meanwhile, the oxygen diffuses inward and react with the substrate to form the ICL. In the ICL, the oxygen partial pressure decreases; thus, Ti reacts with oxygen or water vapor to form Ti_2_O (Figs 4c,d and 6c).


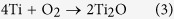



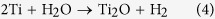


From the resluts of XRD and XPS (Fig. 4), Na_4_Ti_5_O_12_ is formed during the corrosion, and it is enriched on the surface (Fig. 2c) to form part I of the OCL (Fig. 2d). We point out here that Yurkinskii *et al*.^27^ had earlier identified Na_4_Ti_5_O_12_ as the corrosion products of titanium in NaOH melt at 500 °C, while Woo *et al*.^28^ also observed bulk trigonal Na_4_Ti_5_O_12_ via a solid chemical reaction between TiO_2_ and Na_2_CO_3_ at 600 °C. Thus, Na_4_Ti_5_O_12_ could be formed during the corrosion of titanium alloy at 600 °C. Several macro-structural defects (holes and crevice) are present in the OCL for Ti60 alloy specimens exposed in NWO600 for 100 hr. Several authors^1^,^4^,^5^,^7^,^17^ have reported that such macro defects are associated with formation of gaseous corrosion products. In our study, the gaseous products, as well as O_2_ and water vapor, were gathered and dissolved in 500 mL of distilled water. The pH of the resulting solution was about 5 (pH = 5). Shu *et al*.^17^ suggested the primary gaseous products should be TiCl_2_ and HCl. However, TiCl_2_ can not be readily gathered, since it is hyperactive and reacts with water vapor or oxygen easily. Thus, HCl should be produced during corrosion. Thus, the overall reaction now involve Ti oxides, NaCl and water vapor:


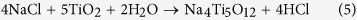


The standard Gibbs free energy change (ΔG°) for reaction (5) is negative, as presented in Table 1, and the reaction is thermodynamically spontaneous. That means the deposited solid NaCl destroys the protective TiO_2_ scale^15^,^16^,^17^.

The produced HCl then reacts with the substrate metal cyclically as follows:


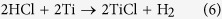



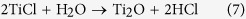



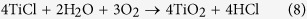



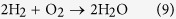


The standard Gibbs free-energy changes (ΔG°) for the above reactions are presented in Table 1. The ΔG° for reaction (6), which is 328 kJ/mol, is positive. However, the ΔG° of the reaction (8) is extremely negative; thus, the HCl/TiCl ratio is very large (about 1.8 × 10^203^ if the partial pressures of H_2_O and O_2_ are 1 atm). Under this condition, the Gibbs free-energy changes (ΔG) of reaction (6) will become −1371 kJ/mol, if the partial pressure of H_2_ is 1 atm. In actual fact, the partial pressures of H_2_O, O_2_ and H_2_ will be much smaller than 1 atm, such that the HCl/TiCl ratio can not be 1.8 × 10^203^. Accordingly, ΔG for reaction (6), though more positive than the calculated value of −1371 kJ/mol, will still be extremely negative. This means that reaction (6) is strongly favored and may actually occur to a signicant extent in our system. A similar situation occurs for reaction (7) even though its ΔG° has not been calculated. Since the corrosion product scale (OCL) is formed by the reaction (7) and reaction (8), which is chemical reaction deposition, the OCL is porous (Fig. 2c,d) and non-protective. On the other hand, the diffusion in the corrosion product scale is repidly as mentioned earlier, and the cyclical reactions could occur continuously, resulting in the fast corrosion of Ti60 alloy under solid NaCl deposit.

At the same time, Al and Zr diffuse outward quickly due to the non-protective corrosion product scale and react directly with oxygen and water vapor during corrosion to form Al-Zr mixed oxides and Al_2_O_3_ which make up part III and the part IV of the OCL respectively as shown in Fig. 5a–c.


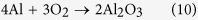



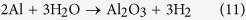



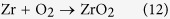



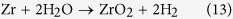


However, Al_2_O_3_ and Al-Zr mixed oxides can not react with solid NaCl and water vapor, since the standard Gibbs free energy changes of reactions (14) and (15) are positive, as presented in Table 1.


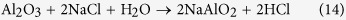



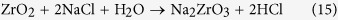


Sn does not diffuse outward and is slightly enriched in the ICL (Fig. 5a,c). After oxygen diffuses into the ICL, Sn reacts with the oxygen in the ICL to form SnO_2_ (Fig. 4d).


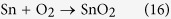


When Ti60 alloy is exposed in NWO600, the metallic elements (Ti, Al and Zr) diffused outwards rapidly to react with the corrosive species (oxygen, water vapor and NaCl) to form the outer corrosion layer (OCL). Whereas, the corrosive species (O, Cl and Na) diffused inwards rapidly and reacted with the substrate to form the inner corrosion layer (ICL).

## Summary

When Ti60 alloy covered with a solid NaCl deposit is exposed in wet oxygen flow at 600 °C, the corrosion scale is complex and divided into outer corrosion layer (OCL) and inner corrosion layer (ICL). The key reaction for the fast corrosion of Ti60 alloy has been modified as the reaction of solid NaCl and Ti oxides rather than the other oxides (like Al oxides or Zr oxides) based on the identification of corrosion products (Na_4_Ti_5_O_12_ and HCl *et al*). This reaction compromises the integrity and protective ability of the corrosion product scale and the resulting corrosion product scale, containing plentiful rapid diffusion channels, is non-protective, thereby aggravating the corrosion process.

## Methods

### Materials preparation

The material used in this study was Ti60 alloy, with chemical composition (mass %) as follows; 5.62 Al; 3.85 Sn; 2.98 Zr; 0.9 Mo; 0.4 Nb; 1.05 Ta; 0.35 Si and balance Ti. The metallographic and the backscattered electron images presented in Fig. 3a,b, respectively show a bimodal microstructure, obtained by near - β forging at about 1035 °C^29^. In Fig. 3b, the β stabilizing elements (Mo, Nb and Ta) are enriched in the write region, and the same enrichment of Zr is also obvious.

The specimens were cut into pieces of 10 mm × 15 mm × 2 mm and ground to 800 grits using silicon carbide papers. Prior to experiments, the specimens were ultrasonically degreased in alcohol for about 20 min and then dried in air. The preheated specimen surfaces were covered with NaCl deposit by repeatedly brushing and drying with saturated NaCl solution^1^,^2^,^3^,^4^,^5^,^6^,^7^,^17^, until about 4 ± 0.2 mg/cm^2^ of NaCl was deposited on the surface.

### Corrosion experiments

**Figure 8:**
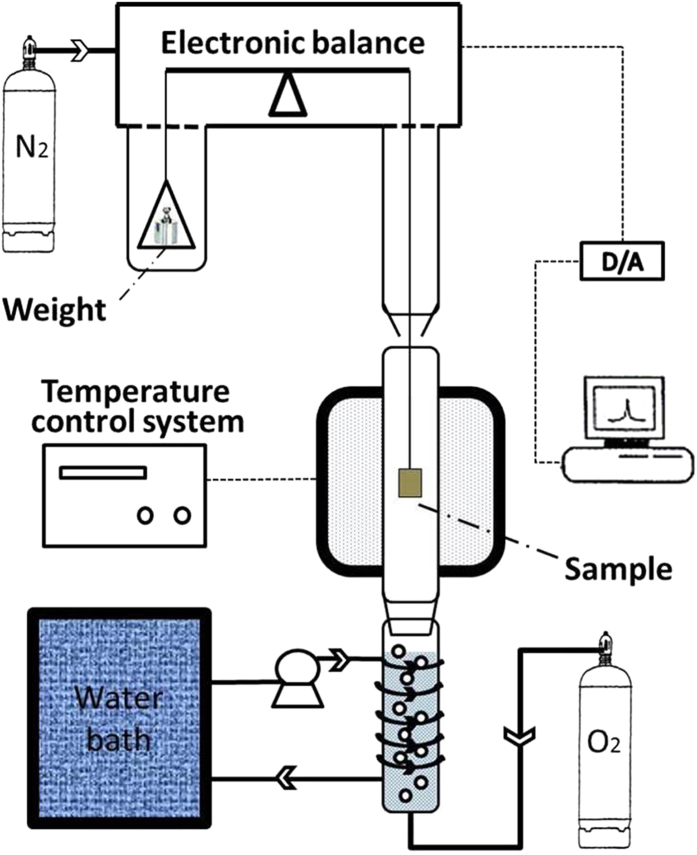
Schematic diagram of the thermo-balance.

Corrosion tests were carried out in a thermo-balance, with schematic diagram as shown in Fig. 8. Thermo-gravimetric analysis (TGA) was adapted to record the continuous mass gain during the corrosion experiment. The test environment (water vapor + O_2_) was obtained by passing pure O_2_ through distilled water by means of a glass bubbler. The volume fraction of water vapor was controlled by adjusting the temperature of the distilled water in the glass bubbler. The concentration of water vapor was about 30.8 vol.%, produced at 70 °C. To prevent the water vapor from condensing inside the thermo-balance, a counter flow of pure N_2_ was passed through the thermo-balance as shown in Fig. 8. The flow rate of N_2_ was 400 mL/min and that of the carrying O_2_ was 140 mL/min (the inner diameter of the tube was about 3.2 cm). After the furnace reached the desired temperature and the gas flows were stabilized, the specimen was quickly lowered into the constant temperature zone of the furnace tube. When the tests were carried out in O_2_ without water vapor, the water in the glass bubbler was removed and the flows of O_2_ and N_2_ were set similar to the moist environment.

In this study, the corrosion behavior of Ti60 alloy was studied in the following corrosion environments: Pure O_2_ flow (O600); wet O_2_ flow (WO600); solid NaCl deposit in pure O_2_ flow (NO600); solid NaCl deposit in wet O_2_ flow (NWO600). The specific environmental parameters are presented in Table 2.

After corrosion, the microstructure morphologies of the corrosion scales were examined by SEM and TEM, and the chemical composition of corrosion products were analyzed in detail by EDS, EPMA, TEM, XPS and XRD.

## Additional Information

**How to cite this article**: Fan, L. *et al*. Corrosion Behavior of Ti60 Alloy under a Solid NaCl Deposit in Wet Oxygen Flow at 600 °C. *Sci. Rep*. **6**, 29019; doi: 10.1038/srep29019 (2016).

## Figures and Tables

**Table 1:** Standard Gibbs free energy changes of reactions at 600°C.

Reaction	ΔG° kJ/mol
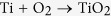	−785
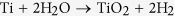	−386
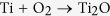	–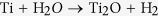	–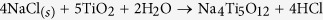	−195
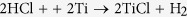	328
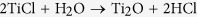	–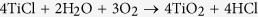	−3397
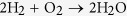	−399
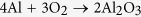	−2804
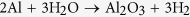	−803
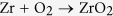	−933
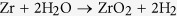	−534
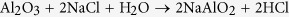	187
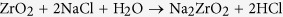	193
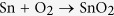	−400

Submitted to ***Scientific Reports*** by Lei Fan *et al*.

**Table 2:** Experimental parameters.

Environment	Mass of NaCl (mg/cm^2^)	Water pressure (kPa)	Flow rate of O_2_^(mL/min)^	^Temperature (˚C)^
NWO600	4.0	31	140	600
NO600	4.0	0	140	600
WO600	0	31	140	600
O600	0	0	140	600

Submitted to *Scientific Reports* by Lei Fan *et al*.
